# Severe sepsis due to severe falciparum malaria and leptospirosis co-infection treated with activated protein C

**DOI:** 10.1186/1475-2875-6-42

**Published:** 2007-04-12

**Authors:** Rajagopala Srinivas, Ritesh Agarwal, Dheeraj Gupta

**Affiliations:** 1 Department of Pulmonary Medicine, Postgraduate Institute of Medical Education and Research, Sector-12, Chandigarh-160012, India

## Abstract

Co-infection with falciparum malaria and leptospirosis is uncommon. The aim of this study is to report a case of severe sepsis secondary to dual infection with falciparum malaria and leptospirosis. The literature is also reviewed on the clinical course of such co-infections, and the possible mechanisms and treatment of patients with life-threatening malaria and leptospirosis with activated protein C. The patient was a 25-year old male admitted in the Respiratory Intensive Care Unit (RICU) with fever, haemolysis, acute renal failure, hepatitis, acute lung injury (ALI) and altered sensorium. A syndromic evaluation was done and investigations revealed falciparum parasitaemia. He was treated with parenteral artesunate, ceftriaxone and doxycycline, and adjunctive therapies as for severe sepsis. Infusion of activated protein C was started 20 hours after onset of organ dysfunction, and intensive haemodialysis was instituted. Over the next four days the patient became afebrile with progressive resolution of ALI, renal failure and hepatitis. His *Leptospira *serology (requested as part of the evaluation) was reported positive on day 5. Dual infections are common and under-recognized in the tropics. Failure to treat potential co-infections may lead to poor outcomes. Acute lung injury in falciparum malaria has high mortality rates and therapy as for severe sepsis may improve survival. Adjunctive therapies, including activated protein C, cannot replace source eradication.

## Background

The world-wide distribution of malaria [1] overlaps with that of other infectious diseases, including leptospirosis. Co-infection of malaria with a wide variety of infectious diseases has been reported. Co-infection of malaria with leptospirosis is rare and has been only been reported in seven (two definite and five probable) cases [2]. Clinical features are unreliable to separate single from dual infections because of the markedly similar clinical syndrome. Patients with dual infections have a severe clinical presentation and their clinical course may be worsened by lack of appropriate therapy for both [2,3]. Patients with severe falciparum malaria, especially those with acute lung injury, have a high mortality rate. Falciparum malaria is due to sequestration of parasites in visceral capillaries with endothelial dysfunction and leptospirosis is an infectious vasculitis; as a result, therapies which improve endothelial function might positively impact the clinical outcomes of both diseases. Newer adjuncts are needed because of the present high mortality rates and the limited benefit conferred by current ancillary therapies. Activated protein C (aPC) has anti-thrombotic, pro-fibrinolytic and anti-inflammatory properties and confers a relative mortality reduction of 22% in patients with severe sepsis and ≥ 2 organ dysfunction [4,5] and has recently been reported to be beneficial in severe falciparum malaria [6,7] and leptospirosis [7]. This is the first reported case of severe sepsis secondary to falciparum malaria with leptospirosis co-infection, with good response to aPC in addition to standard care. Despite the potential benefits of this therapy, source eradication with appropriate antibiotic therapy is paramount. Data on the use of aPC in severe malaria or severe leptospirosis needs to be generated urgently, but ancillary therapies cannot replace the role of syndromic evaluation and therapy.

## Case report

**Figure 1:**
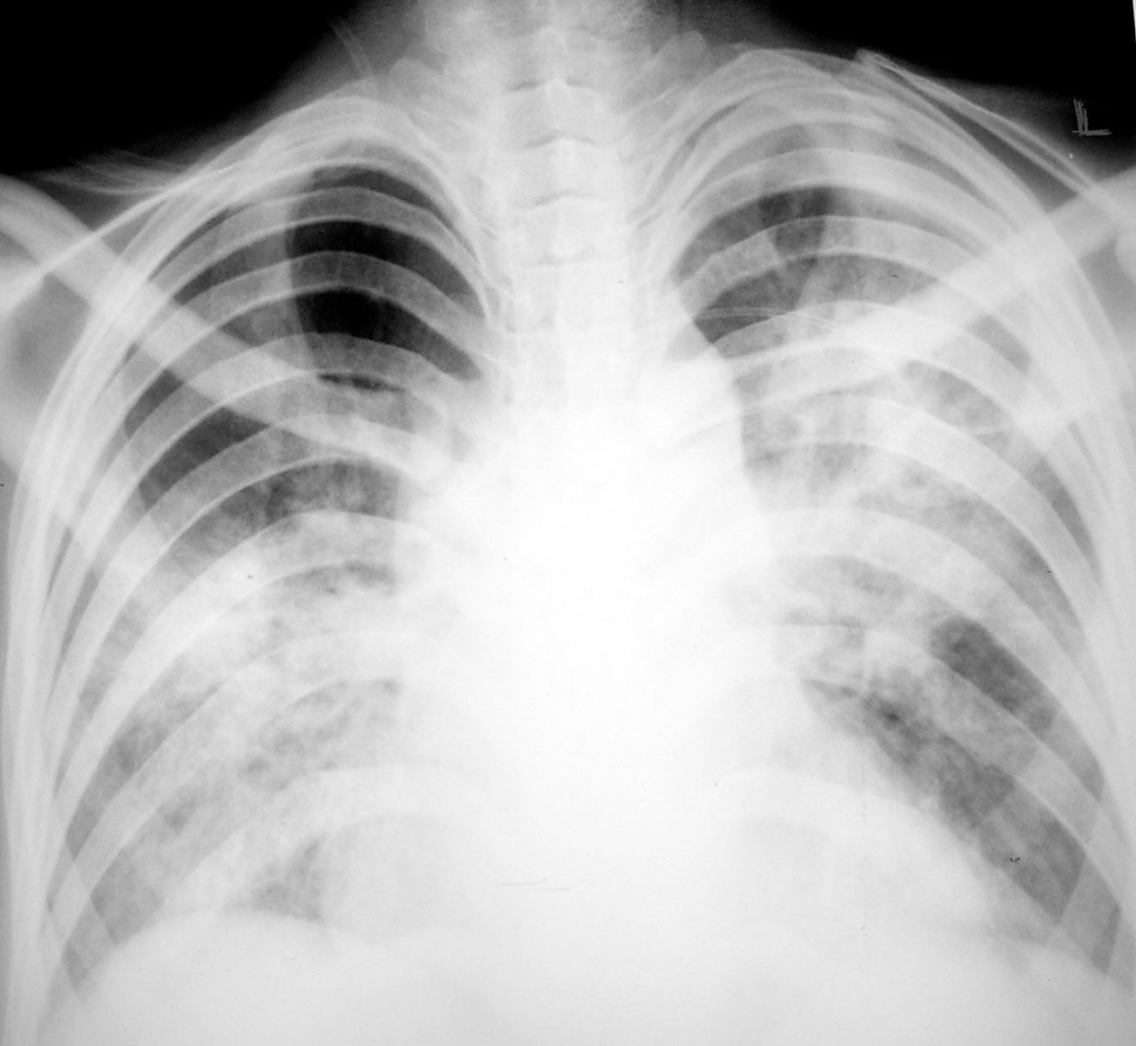
Chest radiograph shows bilateral perihilar alveolar opacities.

A 25-year old male was referred to the Department of Pulmonary Medicine with a short history of high grade fever, myalgia, cola colored urine, decreased urine output and shortness of breath. A clinical diagnosis of severe falciparum malaria (based on respiratory distress, renal impairment and haemoglobinuria) had been considered and he had received two doses of intravenous artesunate (120 mg). On examination he was found to be drowsy and febrile; he also had icterus and sub-conjuctival haemorrhages. The respiratory rate was 45 breaths/minute, blood pressure 110/70 mm Hg and heart rate 126 beats/minute. Auscultation of the chest revealed bilateral crepitations and examination of the abdomen showed hepatosplenomegaly. The rest of the examination was unremarkable. Biochemical investigations revealed a serum creatinine of 7 mg/dL, serum bilirubin 35 mg/dL (conjugated fraction 23 mg/dL), serum bicarbonate of 10 mEq/L, plasma haemoglobin (Hb) 24 mg/dL and urine Hb of 10 mg/dL. Complete blood count showed a haemoglobin of 9.6 gm/dL, total leucocyte count of 15,600/μL and platelet count of 98,000/μL). A syndromic diagnosis of infective hepatorenal syndrome was made and he was managed in the respiratory intensive care unit with artesunate (150 mg o.d), ceftriaxone (two gm b.i.d) and doxycycline (100 mg b.i.d), stress ulcer and deep venous thrombosis prophylaxis and intensive blood glucose control. A thin blood smear was positive for falciparum ring forms (1.2%) and gametocytes (with reduction in parasitaemia possibly due to artesunate given empirically); an assay for histidine-rich protein 2 obtained simultaneously was also positive. A clotting profile was normal and his arterial blood gas revealed combined metabolic and respiratory acidosis (pH 7.12) and hypoxaemia with a PaO_2_/FiO_2 _score of 205. Chest X-ray revealed bilateral peri-hilar infiltrates suggestive of acute lung injury (Figure 1). His blood cultures, urine cultures, HEV serology and Weil-Felix test were negative. Intensive haemodialysis was instituted for acute renal failure. In view of severe sepsis and multi-organ dysfunction, an infusion of activated protein C was also started 20 hours after the onset of organ dysfunction. The patient gradually improved and at 96 hours he was afebrile, alert with progressive resolution of acute lung injury (ALI) and hepatitis (Table 1). He remained oliguric and haemodialysis was continued. IgM ELISA for leptospira (sent as part of the evaluation) was reported positive on day 5. Artesunate, ceftriaxone and doxycycline were continued for seven days. His renal functions started improving on day 10 and dialysis was discontinued. His hospital course was complicated by right peroneal palsy which improved with physiotherapy. He was discharged on day 14 of hospital admission. Patient was asymptomatic at two month follow-up.

**Table 1:** Physiological and clinical variables during first 7 days of RICU stay demonstrating the effect of the infusion of Drotrecogin alfa (activated) on days 1–4.

Variables	Day 0	Day 1	Day 2	Day 3	Day 4	Day 5	Day 6	Day 7
Heart rate (/minute)	128	111	120	116	106	100	92	86
Respiratory rate (/minute)	46	32	36	30	28	28	24	22
Temperature (C)	39	38.5	38.4	38	37	37	37	37.2
pH	7.2	7.25	7.26	7.3	7.34	7.42	7.44	-
PaO_2_/FiO_2_	168	180	130	230	260	290	340	-
HCO_3 _(mEq/L)	13	15	14	18	20	22	22	-
Ventilatory mode	Spontaneous	ACMV	ACMV	ACMV	PSV	T-piece	Spontaneous	Spontaneous
PEEP (cm H_2_0)	-	8	10	6	5	-	-	-
Serum creatinine (mg/dL)	5.2	6.0	5.2	7.1	4.6	4.4	4.0	3.6
Urine output (mL/24 hours)	460	100	110	200	240	200	270	560
Serum bilirubin (mg/dL)	18.2	18.9	18.2	15.4	9.1	8.4	6.0	4.2
Total leucocyte count (/μL)	19000	19500	18200	14400	11400	8100	8000	8100
Platelet count (/μL)	92000	105,000	155,000	183,000	212,000	241,000	260,000	270,000

## Discussion

There are about 500 million estimated cases of malaria, with 2–3 million severe malaria cases and 1.1 million deaths each year worldwide [8]. Leptospirosis is an increasingly recognized infection in the tropics. Presence of the causative agent, farming, poor sanitation and hygiene provide the substrate for the observed seasonal epidemics. However, the infection is under-recognized because most infections are anicteric, resolve spontaneously and mimic other febrile illnesses.

In a patient with fever, renal failure and jaundice the differential diagnosis include severe malaria, severe leptospirosis, enteric fever, Hantaan virus, viral hepatitis with fulminant liver failure and scrub typhus [9,10]. The local epidemiology, presence of haemolysis and lung injury in this patient restricted the differential diagnosis to malaria, leptospirosis, typhoid and viral hepatitis (especially HEV related). These entities may be impossible to resolve clinically and empiric therapy to cover all of these is justified initially because of the turn-around time of investigations in the tropics. The patient was started on artesunate, ceftriaxone and doxycycline accordingly. In a patient with renal failure and hyperparasitaemia, artesunate has the advantage of rapidity of action, smaller infusion volumes, lesser toxicity and a mortality reduction of 34.7%[11]. In patients with severe leptospirosis, doxycycline is as efficacious as penicillin[12] and is therapeutic for scrub typhus as well.

As malaria and leptospirosis are common in the tropics, co-infections are expected to be common. However such a scenario has seldom been reported [2,13]. This may be due to the unavailability of assays for leptospirosis in resource-constrained areas and failure to anticipate co-infections because of the overlapping clinical features. A significant number of unanticipated co-infections have been reported when routine syndromic serologic panels were employed [13]. Failure to recognize acute leptospirosis co-infection causes delay in the initiation of directed therapy and potentially preventable excess mortality. IgM ELISA antibodies become detectable by the end of first week and have replaced MAT (macro-agglutination test) for routine diagnosis because of the early seroconversion, availability of commercial bedside kits and excellent sensitivity and specificity [14]. Although this patient had a modified Faine score [15] of 31 (with > 25 presumptive of leptospirosis), the validity of this score in co-infections has not been evaluated. Given the clinical features and high specificity of IgM ELISA and unavailability of MAT, the diagnosis of leptospirosis is highly probable.

Acute respiratory distress syndrome in falciparum malaria has an incidence of 21% in those with severe malaria requiring hospitalization [16]. Mortality may be as high as 30% in this group and a high frequency of bacterial super-infections occur [17]. Pulmonary lesions in leptospirosis are primarily haemorrhagic with an incidence from 20–70%. The extent of involvement may have no relation to the presence of icterus and may be self-limited or extensive and fatal.

In patients with severe falciparum malaria, extensive organ sequestration may lead to endothelial clogging and dysfunction. Microthrombosis and vasospasm due to endothelial dysfunction lead to organ hypo-perfusion and worsening lactic acidosis. Adjunctive therapies like exchange transfusion [18] and anti-oxidants have shown no survival advantage [19]. Leptospirosis is an infectious vasculitis with the potential to accentuate endothelial dysfunction in co-infections. At the time of evaluation of this patient, a MEDLINE search and communication with manufacturers (Eli Lilly) revealed no reported data on the use of aPC in malaria or leptospirosis. Subsequently, five cases of successful use of aPC in severe malaria and one in leptospirosis has been reported [6]. This remains the first report of complicated dual infection treated with aPC. As the parasitaemia cleared in less than 36 hours of onset of illness (due to early artesunate institution) exchange transfusion was not contemplated [18]. He had worsening organ dysfunction and was initiated on an infusion of drotrecogin alpha 24 μg/kg/hr for 96 hours and antibiotics. Initiation was guided by the time to establishment of organ(s) failure and was before 48 hours from the first organ failure [4]. Drotrecogin alfa treatment has been shown to reduce mortality in patients with severe sepsis and has been approved for the treatment of patients with severe sepsis who have two or more organ dysfunction and/or APACHE II scores more than 25 [4], and may have a role in complicated tropical infections as well [7]. An improvement in respiratory function and more rapid resolution of cardiovascular dysfunction has been demonstrated. Most of these activities appear to involve the modulation of endothelial function, modulation of leukocyte activity, and improvement in microvascular perfusion in severe sepsis, thus improving organ function [20]. In contrast to sepsis of other aetiologies, the intense endothelial clogging by malaria and denudation due to leptospirosis may have contributed to a slower response. A similar delayed response has been observed in another reported case [6]. Large trials of use of aPC in malaria and leptospirosis, though urgently needed, are unlikely to be conducted given the costs involved. Lack of evidence of benefit is not evidence of lack of benefit and the use of aPC in severe falciparum malaria and leptospirosis is justified pending large trials for the same. Source control represents a key component of success therapy in sepsis and is the best way to quickly reduce parasitic or bacterial load [21]. The appropriate institution of antimicrobials guided by clinical judgment is thus paramount and cannot be replaced by ancillary therapies.

## Conclusion

Dual infections are common and under-recognized in the tropics. Clinical features are unreliable to separate single infections from dual infections, which may have a severe clinical course. Both falciparum malaria and leptospirosis are associated with significant endothelial dysfunction and therapies that improve endothelial function might positively impact clinical outcomes of both diseases. aPC has anti-thrombotic, pro-fibrinolytic and anti-inflammatory properties which can improve endothelial function. Despite the potential benefits of this therapy, source eradication with appropriate antibiotic therapy is paramount. Data on the use of aPC in severe malaria or severe leptospirosis needs to be generated urgently but the role of syndromic evaluation and empiric therapy must not be relegated.
